# Towards standardization of absolute SPECT/CT quantification: a multi-center and multi-vendor phantom study

**DOI:** 10.1186/s40658-019-0268-5

**Published:** 2019-12-26

**Authors:** Steffie M. B. Peters, Niels R. van der Werf, Marcel Segbers, Floris H. P. van Velden, Roel Wierts, Koos (J.) A. K. Blokland, Mark W. Konijnenberg, Sergiy V. Lazarenko, Eric P. Visser, Martin Gotthardt

**Affiliations:** 1 Department of Radiology and Nuclear Medicine, Radboudumc, P.O. Box 9101, 6500 HB Nijmegen, The Netherlands; 2 Department of Radiology and Nuclear Medicine, Erasmus MC, Rotterdam, The Netherlands; 3 Department of Medical Physics, Albert Schweitzer Hospital, Dordrecht, The Netherlands; 4 Department of Radiology, Section of Medical Physics, Leiden University Medical Center, Leiden, The Netherlands; 5 Department of Radiology and Nuclear Medicine, Maastricht UMC+, Maastricht, The Netherlands; 6 Department of Nuclear Medicine, Noordwest Ziekenhuisgroep, Alkmaar, The Netherlands

**Keywords:** SPECT/CT, absolute quantification, recovery coefficient, performance evaluation

## Abstract

**Abstract:**

Absolute quantification of radiotracer distribution using SPECT/CT imaging is of great importance for dosimetry aimed at personalized radionuclide precision treatment. However, its accuracy depends on many factors. Using phantom measurements, this multi-vendor and multi-center study evaluates the quantitative accuracy and inter-system variability of various SPECT/CT systems as well as the effect of patient size, processing software and reconstruction algorithms on recovery coefficients (RC).

**Methods:**

Five SPECT/CT systems were included: Discovery™ NM/CT 670 Pro (GE Healthcare), Precedence™ 6 (Philips Healthcare), Symbia Intevo™, and Symbia™ T16 (twice) (Siemens Healthineers). Three phantoms were used based on the NEMA IEC body phantom without lung insert simulating body mass indexes (BMI) of 25, 28, and 47 kg/m^2^. Six spheres (0.5–26.5 mL) and background were filled with 0.1 and 0.01 MBq/mL ^99m^Tc-pertechnetate, respectively. Volumes of interest (VOI) of spheres were obtained by a region growing technique using a 50% threshold of the maximum voxel value corrected for background activity. RC, defined as imaged activity concentration divided by actual activity concentration, were determined for maximum (RC_max_) and mean voxel value (RC_mean_) in the VOI for each sphere diameter. Inter-system variability was expressed as median absolute deviation (MAD) of RC. Acquisition settings were standardized. Images were reconstructed using vendor-specific 3D iterative reconstruction algorithms with institute-specific settings used in clinical practice and processed using a standardized, in-house developed processing tool based on the SimpleITK framework. Additionally, all data were reconstructed with a vendor-neutral reconstruction algorithm (Hybrid Recon™; Hermes Medical Solutions).

**Results:**

RC decreased with decreasing sphere diameter for each system. Inter-system variability (MAD) was 16 and 17% for RC_mean_ and RC_max_, respectively. Standardized reconstruction decreased this variability to 4 and 5%. High BMI hampers quantification of small lesions (< 10 ml).

**Conclusion:**

Absolute SPECT quantification in a multi-center and multi-vendor setting is feasible, especially when reconstruction protocols are standardized, paving the way for a standard for absolute quantitative SPECT.

## Introduction

Accurate absolute quantification of radiotracer distribution is essential for dosimetry aimed at personalized radionuclide therapy and may improve prediction of therapy response, prevention of toxicity effects, and treatment follow-up [1, 2]. Both positron emission tomography (PET) and single-photon emission computed tomography (SPECT) hold the promise for absolute radioactivity quantification. However, for SPECT, quantification is considered less straightforward [3, 4] since its accuracy depends on a variety of factors, including the necessary use of a collimator, the varying detector trajectory, and the need for more complicated scatter correction and attenuation correction than in PET [4]. Furthermore, quantification is influenced by both the reconstruction algorithm and settings. Recent developments in corrections for photon attenuation and scatter, collimator modeling and 3D reconstruction, e.g., by including resolution recovery and noise regulation, have improved reconstruction techniques, thereby enabling absolute SPECT quantification [5]. The addition of an integrated computed tomography (CT) system not only provides an anatomical reference but enables accurate attenuation and scatter correction as well, improving quantification [6]. Nowadays, combined SPECT/CT systems have become standard clinical practice.

Standardization of protocols in such a way that quantitative results can be reliably compared between systems requires more insight in their quantitative accuracy and performance. For PET/CT, differences in absolute quantification of various systems have been extensively characterized through the European Association of Nuclear Medicine initiative of EANM Research Ltd. (EARL). As part of this initiative, quantification of the most widely used PET radiotracer, ^18^F-fluorodeoxyglucose (^18^F-FDG), has been standardized in a multi-center setting through an accreditation program [7, 8].

Until date, no similar efforts for SPECT/CT have been carried out, which hampers multi-center research trials involving absolute SPECT quantification, especially those aimed towards dosimetry. The requirements on quantification for dosimetry are described in MIRD Pamphlet No. 23 [9]. With the advent of, for example, ^177^Lu-PSMA therapy [10–13], it is expected that dosimetry will play a pivotal role for reliable determination of dose response relationships. But also our understanding of biomarker studies and already well-established radionuclide therapies in thyroid cancer [14, 15] or neuroendocrine tumors [16–20] may profit from optimized quantitative SPECT imaging for sophisticated dosimetry. In addition, quantitative measurements are increasingly used in diagnosis or disease monitoring [21]. Several studies investigated the quantitative performance of SPECT for a variety of radionuclides, including technetium-99m (^99m^Tc) [22, 23], indium-111 (^111^In) [24–26], iodine-131 (^131^I) [27], lutetium-177 (^177^Lu) [28], yttrium-90 (^90^Y) [29], or a combination of these [30, 31]. However, comparing these results of absolute quantification may be difficult as they were obtained on different SPECT/CT systems. Seret et al. [32] compared four SPECT/CT systems for their quantitative capabilities and found that for objects which dimensions exceeded the SPECT spatial resolution several times, quantification was possible within a 10% error. For smaller structures, larger errors were observed necessitating partial volume effect correction. Furthermore, reconstruction artifacts degraded the accuracy of quantification. Hughes and colleagues compared image quality [33] of three SPECT/CT systems for cardiac applications. They showed that these systems performed differently in terms of quantitative accuracy, contrast, signal-to-noise, and uniformity. In a different study [34] in which they compared the same three SPECT/CT systems, they showed that image resolution is very much dependent on the reconstruction algorithm. In recent years, various SPECT/CT and software vendors have responded to the increasing need for SPECT quantification and now commercially offer software packages for quantification of several radionuclides including ^99m^Tc, ^111^In, ^131^I, and ^177^Lu [35–38].

The aim of this study is to compare absolute quantification for state-of-the-art SPECT/CT systems from different vendors at different imaging centers for ^99m^Tc. Multiple quantitative reconstruction algorithms that are currently commercially available are included in the comparison. The quantitative accuracy and inter-system variability of recovery coefficients (RC) are determined using various phantom experiments. The effects of lesion volume, patient size, reconstruction algorithm, and post-processing on RC are investigated. The results of these comparisons provide a first step towards a vendor-independent standard for absolute quantitative SPECT/CT that would allow transferability of the obtained metrics [39].

## Methods

### SPECT/CT systems

Data were acquired on five state-of-the-art SPECT/CT systems from three manufacturers: a Discovery NM/CT 670 Pro (GE Healthcare, Milwaukee, USA), a Precedence 6 (Philips Healthcare, Best, The Netherlands), a Symbia Intevo 6, and two Symbia T16’s (Siemens Healthineers, Erlangen, Germany) (Table 1).

**Table 1:** Characteristics of all used SPECT/CT systems with LEHR collimator

System	Discovery NM/CT 670 Pro	Precedence 6	Symbia Intevo 6	Symbia T16
Detector crystal	3/8” NaI	3/8” NaI	3/8” NaI	3/8” NaI
PMT*	59	55	59	59
FOV*	40 × 54 cm	38.1 × 50.8 cm	38.7 × 53.3 cm	38.7 × 53.3 cm
Hole shape	Hexagonal	Hexagonal	Hexagonal	Hexagonal
Number of holes (× 1000)	Not specified	86.4	148	148
Collimator hole diameter	1.50 mm	1.40 mm	1.11 mm	1.11 mm
Hole length	35 mm	32.8 mm	24.05 mm	24.05 mm
Septal thickness	0.2 mm	0.152 mm	0.16 mm	0.16 mm
Sensitivity for ^99m^Tc @ 10 cm	72 cps/MBq	66 cps/MBq	91 cps/MBq	91 cps/MBq
Septal penetration @ 140 keV	0.3%	1.3%	1.5%	1.5%
Planar resolution^†^	7.4 mm	7.4 mm	7.5 mm	7.5 mm
SPECT central resolution^†^	6.4 mm	4.4 mm	4.4 mm	4.4 mm
SPECT peripheral radial resolution^†^	5.7 mm	4.2 mm	4.0 mm	4.0 mm
SPECT peripheral tangential resolution^†^	5.1 mm	4.3 mm	3.9 mm	3.9 mm

* (*C*)*FOV* (center) field of view, *PMT* photomultiplier tube

† Spatial resolution without scatter (LEHR collimator at 10 cm, (full width at half maximum (FWHM) in CFOV [mm], 3/8” crystal)

### Phantoms

**Fig. 1:**
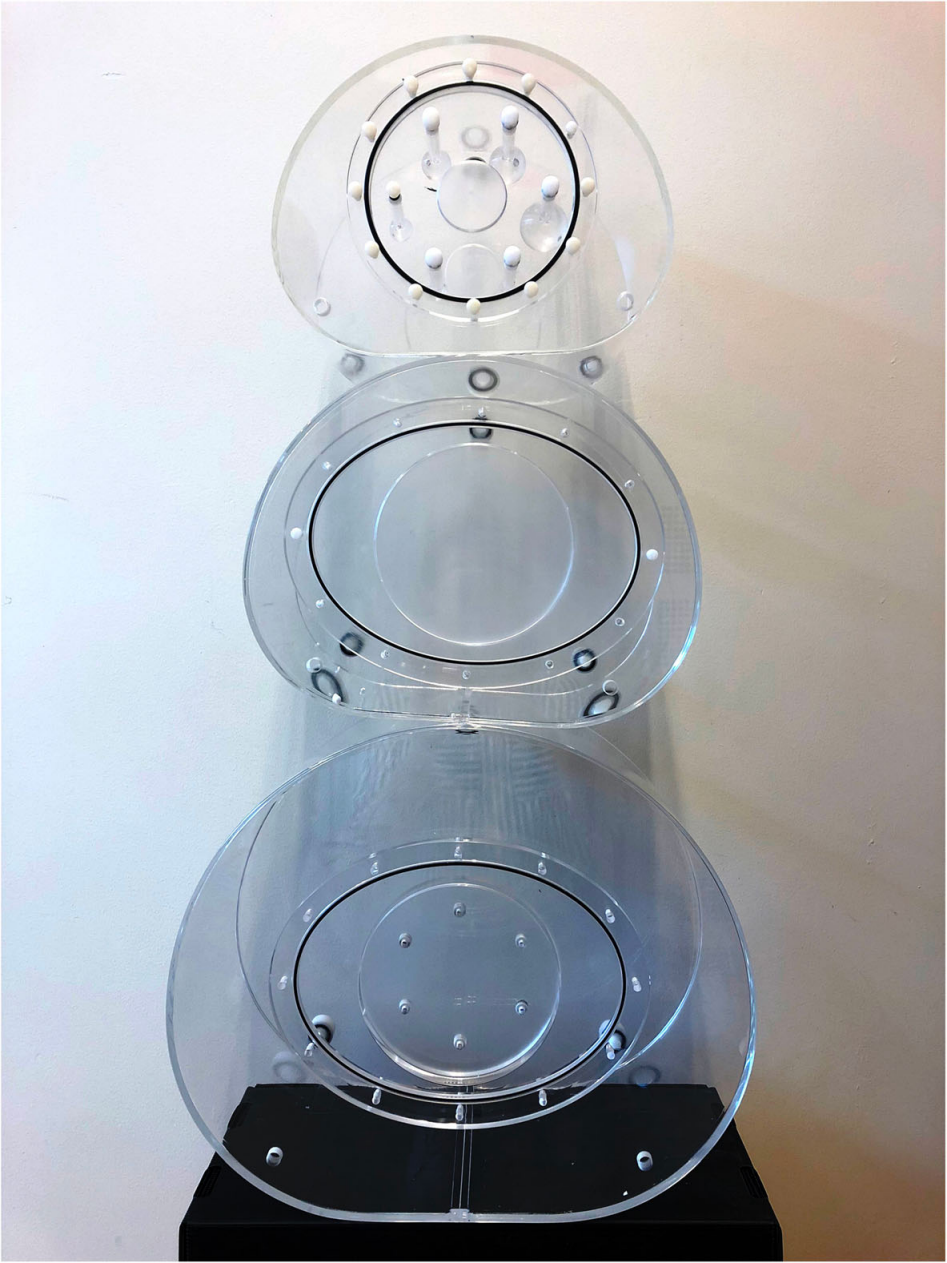
The phantoms used to determine the RC. Upper phantom: NEMA IEC body phantom. Lower two phantoms: custom-made phantoms reflecting a larger body mass index (BMI, kg/m^2^) of patients. Note that the lower two phantoms are depicted without spheres inset

A NEMA IEC body phantom without lung insert was used (Fig. 1). This phantom represents a patient with a body mass index (BMI) of 25 kg/m^2^ (which is considered normal) and contains six spheres with inner diameters (and corresponding volumes) of 10 mm (0.5 ml), 13 mm (1.2 ml), 17 mm (2.6 ml), 22 mm (5.6 ml), 28 mm (11.5 ml), and 37 mm (26.5 ml). To evaluate the effect of patient size on SPECT quantification, two additional custom-made phantoms were used on some systems that were similar to the shape of the NEMA IEC body phantom, but with larger diameters, reflecting a larger BMI of obese patients (Table 2). The spheres from the NEMA IEC body phantom were also used for the increased body size phantoms.

**Table 2:** Phantom sizes and corresponding patient characteristics

Phantom	Volume (l)	Waist circumference (cm)	Corresponding patient BMI* (kg/m^2^)
Small (NEMA phantom)	9.70	85	25
Medium	14.73	100	28
Large	25.96	130	47

* *BMI* body mass index

For all phantoms, the spheres and background compartment were filled with a homogeneous solution of ^99m^Tc-pertechnetate in water with a concentration of approximately 100 kBq/ml and 10 kBq/ml, respectively, resulting in a sphere-to-background ratio of 10:1 similar to EARL guidelines for ^18^F-FDG PET imaging [8]. All ^99m^Tc-pertechnetate activities were measured in the clinical radionuclide dose calibrators present in the participating hospitals, which undergo regular quality control according to national guidelines [40].

### Data acquisition and reconstruction

Harmonized acquisition protocols were used for all measurements. Images were acquired with a low-energy high-resolution (LEHR) collimator (Table 1) in step and shoot mode, 128 projections (64 per detector head) (Discovery NM/CT 670 Pro: 120 projections, 60 per detector head), 20 s per projection, zoom factor 1.0, matrix size 128 × 128 (Symbia Intevo, 256 × 256), a photon energy window of 140 keV ± 15% and the detector trajectory set to body contour. Data from the standard NEMA phantom were acquired five times repetitively to assess system-specific repeatability. The time per angle was adjusted to obtain similar count statistics for each replicate.

Data were reconstructed with two reconstruction methods to assess its influence on quantification. First, vendor-specific 3D iterative reconstruction algorithms that included scatter correction, CT-based attenuation correction (for acquisition parameters see Additional file 1: Table S1) and resolution recovery with institute-specific settings used in clinical practice [3] were used. This included two quantitative reconstruction algorithms that are currently commercially available (GE Q.Metrix and Siemens xSPECT Quant). Second, data were reconstructed with a vendor-neutral quantitative reconstruction algorithm (Hybrid Recon v1.1.2; Hermes Medical Solutions, Stockholm, Sweden) (Table 3).

**Table 3:** Reconstruction and quantification parameters and processing software used in this study

System	Discovery NM/CT 670 Pro	Precedence 6	Symbia Intevo 6	Symbia T16 system 1	Symbia T16 system 2	All
Imaging center	Leiden University Medical Center	Maastricht University Medical Center	Noord West ziekenhuis Groep	Radboud University Medical Center	Erasmus University Medical Center	All
Reconstruction	OSEM* + Evolution with PSF* correction	OSEM* + Astonish with PSF* correction	Weighted Conjugate Gradient + xSPECT with PSF* correction	OSEM* + Flash 3D with PSF* correction	OSEM* + Hybrid Recon V1.2 with PSF* correction	OSEM* + Hybrid Recon V1.2 with PSF* correction
Quantification	Q.Metrix	Manual analysis	xSPECT Quant	Manual analysis	Hermes SUV SPECT	Hermes SUV SPECT
Iterations	9 [5]	3	24	6	5	5
Subsets	10	16	2	16	16	16
Post-reconstruction filter	None	None	7.5 mm (Gaussian)	8.4 mm (Gaussian)	5 mm (Gaussian)	5 mm (Gaussian)
Processing	GE Xeleris 4.0 workstation	Philips Extended Brilliance Workspace	Siemens Syngo.via	Siemens Inveon Research Workplace	Hermes Hybrid Viewer	In-house developed Python algorithm
Attenuation correction	CT-based, bilinear conversion of HU into attenuation coefficients at 140 keV	CT-based, HU segmentation using a step-like law, bilinear conversion of HU into attenuation coefficients at 140 keV	CT-based, bilinear conversion of HU into attenuation coefficients at 140 keV	CT-based, bilinear conversion of HU into attenuation coefficients at 140 keV	CT-based, Bilinear conversion of HU into attenuation coefficients at 140 keV	CT-based, bilinear conversion of HU into attenuation coefficients at 140 keV
Scatter Correction	DEW* (120 keV ± 10%)	Kernel based	DEW* (119 keV ± 7.5%)	DEW* (119 keV ± 10%)	Monte Carlo-based	Monte Carlo-based
Image voxel size	2.21 × 2.21 × 2.21 mm^3†^	4.7 × 4.7 × 4.7 mm^3^	2.54 × 2.54 × 2.54 mm^3^	4.8 × 4.8 × 4.8 mm^3^	4.8 × 4.8 × 4.8 mm^3^	4.8 × 4.8 × 4.8 mm^3^

* OSEM ordered subset expectation maximization, *PSF* point spread function, *DEW* dual energy window

† Initial acquisition was performed with 128 × 128 matrix size and corresponding voxel size of 4.42 × 4.42 × 4.42 mm^3^. For quantification purposes this was interpolated to a 256 × 256 matrix size and corresponding voxel size of 2.21 × 2.21 × 2.21 mm^3^, as recommended by the vendor.

### Calibration factor

SPECT/CT systems were cross-calibrated for ^99m^Tc with the corresponding dose calibrators according to the manufacturer’s recommendation or to the center’s standard practice (Additional file 1: Table S2). Either one large or multiple smaller cylindrical regions of interest (ROIs) where drawn to obtain a calibration factor (CF) according to:

1 $$ \mathrm{CF}\ \left[\frac{\mathrm{cps}/\mathrm{ml}}{\mathrm{kBq}/\mathrm{ml}}\right]=\frac{\left(\frac{\mu\ }{t\bullet n\bullet \nu}\right)}{A} $$

where *μ* is the mean voxel value in the reconstructed image, *t* is the time per projection, *n* is the number of projections, *ν* is the voxel size, and *A* is the actual activity concentration in the phantom.

### Analysis

To evaluate the absolute quantification of different SPECT/CT systems, RC for background and all six spheres were determined. RC was defined as the ratio of the measured activity concentration (*a*) and the true activity concentration (*A*) for each sphere:

2 $$ \mathrm{RC}=\frac{a}{A} $$

Volumes of interest (VOIs) for each sphere were determined with a region growing algorithm for which the cut-off threshold was calculated by [41]:

3 $$ {\mathrm{VV}}_{\mathrm{thresh}}=0.5\bullet \left({\mathrm{VV}}_{\max, \mathrm{sphere}}+{\mathrm{VV}}_{\mathrm{mean},\mathrm{bg}}\right) $$

where VV_thresh_ is the threshold voxel value, VV_max,sphere_ is the maximum voxel value in the sphere VOI, and VV_mean,bg_ is the mean voxel value in the background VOI. VV_mean,bg_ was determined by placing six cylindrical VOIs (diameter 4–5 cm) in a uniform region within the phantom.

The maximum and mean activity concentration for each sphere were determined, which resulted in both maximum and mean RC values, denoted as RC_max_ and RC_mean_, respectively.

The repeatability of the RC for each system was assessed with the reconstructed data of the five repetitive measurements by calculating the median absolute deviation (MAD) for each sphere diameter according to:

4 $$ \mathrm{MAD}=\mathrm{median}\left(\left|{\mathrm{RC}}_i-\overset{\sim }{\mathrm{RC}}\right|\right) $$

where RC_*i*_ is the recovery coefficient of measurement *i* and $\overset{\sim }{\mathrm{RC}}$ is the median recovery coefficient of all repetitive measurements.

The MAD was also used to assess variability between systems for each sphere diameter. For each sphere, the median RC from each system was used in Eq. 4. This resulted in a sphere-specific MAD.

In addition to center-specific image analysis, all images were processed automatically in a standardized way using in-house developed software in Python which uses the SimpleITK toolkit region growing algorithm to determine sphere-specific VOIs using the same region growing algorithm as described above (Table 4) [42, 43].

**Table 4:** Calibration factors for center-specific and vendor-neutral reconstructions, calculated for 128 projections and 20 s/projection

System	Center-specific CF* (cps/kBq)	CF for Hermes SUV SPECT (kBq/cts)
Discovery NM/CT 670 Pro	0.075	0.128
Precedence 6	0.0986	0.143
Symbia Intevo 6	1.00 [-]^†^	0.112
Symbia T16 system 1	0.0951	0.114
Symbia T16 system 2	0.110	0.110

* *CF* calibration factor

† Data is already quantitative in kBq therefore no calibration factor is stated

## Results

### Calibration factor

The calibration factors that were used to determine the RC for each system can be found in Table 4.

### Recovery coefficient

Differences (indicated as mean ± standard deviation) between the RC determined using standardized processing software versus center-specific processing software were 2 ± 3% for RC_mean_ and 0 ± 3% RC_max_. Since these differences were considered negligible, all data were processed using the standardized processing software (Python) as described earlier (performed centralized by two authors on all data).

**Fig. 2:**
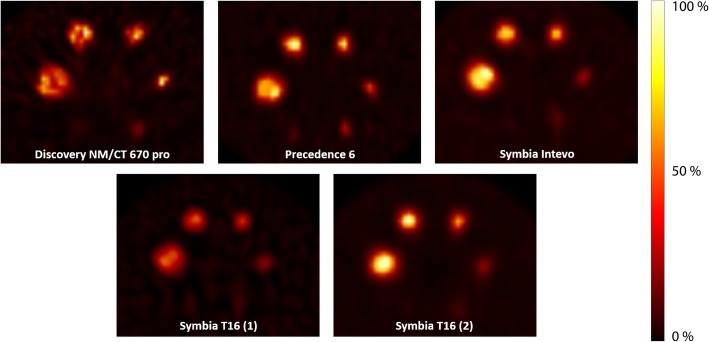
Images of the NEMA IEC body phantom for all systems, reconstructed with a vendor-specific algorithm

The median recovery coefficient of the background compartment of the phantom was 1.01 (range, 0.93–1.07). The sphere-to-background activity concentration ratio was 10.6 ± 0.4:1 for all systems. Images obtained on all five systems showed different visual results (Fig. 2).

**Fig. 3:**
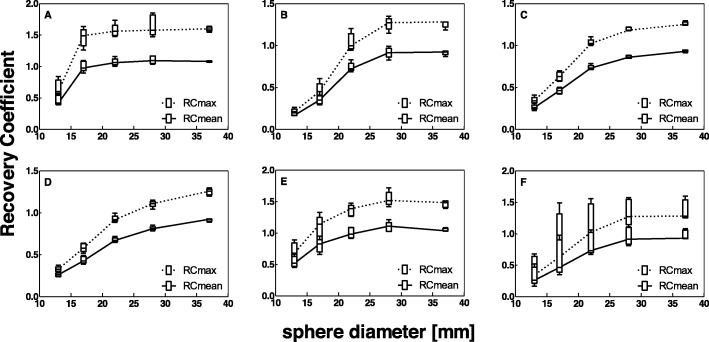
Recovery coefficient as a function of sphere diameter for all systems separately (**a**–**e**) and for all systems combined (**f**), for data reconstructed with a vendor-specific algorithm. Median and box plot for five repetitive measurements per system. (**a**) GE Discovery NM/CT 670 Pro, (**b**) Philips Precedence 6, (**c**) Siemens Symbia Intevo 6, (**d**) Siemens Symbia T16 system 1, (**e**) Siemens Symbia T16 system 2, (**f**) Median RC values for all systems combined

For all systems, both RC_mean_ and RC_max_ decreased with decreasing sphere diameter (Fig. 3a–e). RC for the smallest sphere diameter (10 mm) could not be obtained because of the low contrast between the smallest sphere and the background for the used activity concentration ratio. Therefore, this sphere diameter is not considered in the remainder of this study. The variability in RC between systems is visualized in Fig. 3f.

For each system, RC repeatability, expressed as the MAD, was best for the largest spheres, but good repeatability was shown for all sphere diameters (Table 5).

**Table 5:** MAD per system (median and range over all sphere diameters) for data reconstructed using a vendor and center-specific algorithm

	RC_mean_	RC_max_
Discovery NM/CT 670 Pro	0.02 (0.01—0.08)	0.06 (0.02—0.10)
Precendence 6	0.02 (0.00—0.04)	0.03 (0.00—0.06)
Symbia Intevo 6	0.01 (0.01—0.03)	0.01 (0.00—0.05)
Symbia T16 (1)	0.02 (0.00—0.04)	0.02 (0.01—0.05)
Symbia T16 (2)	0.07 (0.00—0.09)	0.09 (0.03—0.19)

### Effect of reconstruction algorithm on RC

**Fig. 4:**
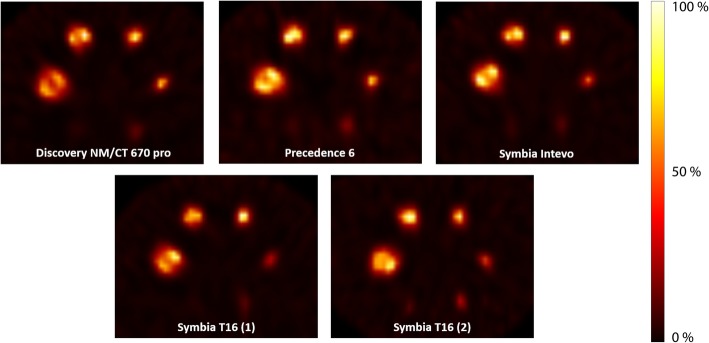
Images of the NEMA IEC body phantom for all systems, reconstructed with a vendor-neutral algorithm

**Fig. 5:**
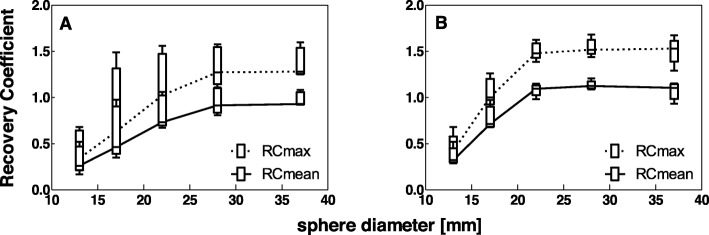
Recovery coefficient for all systems combined as a function of sphere diameter for vendor-specific reconstruction (**a**) and vendor-neutral reconstruction (**b**)

Vendor-neutral reconstruction showed a large decrease in inter-system variability (Figs. 4 and 5). This finding is further confirmed by the MAD for reconstruction with vendor-specific versus vendor-neutral software (Table 6), which shows a median MAD of 0.10 and 0.17 (16 and 17%) for the RC_mean_ and RC_max_ of vendor-specific reconstruction, and a decreased median MAD of 0.04 and 0.05 (4 and 5%) for the RC_mean_ and RC_max_ of vendor-neutral reconstruction, respectively.

**Table 6:** MAD per sphere diameter for all systems combined, using either vendor-specific or vendor-neutral reconstruction algorithms.

Sphere diameter	RC_mean_		RC_max_	
	Vendor-specific	Vendor-neutral	Vendor-specific	Vendor-neutral
37 mm	0.01	0.05	0.03	0.05
28 mm	0.10	0.02	0.16	0.04
22 mm	0.20	0.04	0.28	0.06
17 mm	0.11	0.04	0.18	0.11
13 mm	0.09	0.04	0.13	0.04

### Effect of patient size on RC

**Fig. 6:**
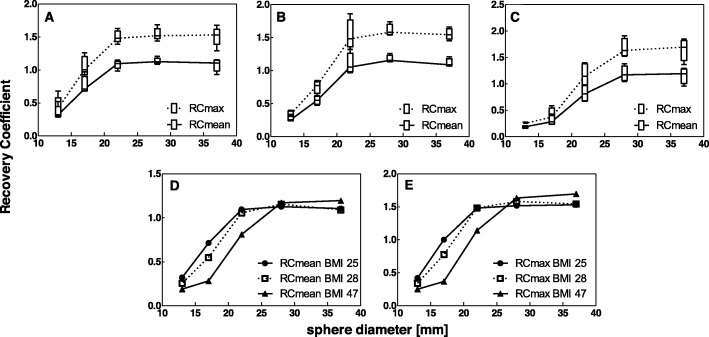
RC per sphere diameter for (**a**) small phantom (BMI, 25 kg/m^2^), (**b**) medium phantom (BMI, 28 kg/m^2^), (**c**) large phantom (BMI, 47 kg/m^2^), (**d**–**e**) RC_mean_ and RC_max_ for all three phantom volumes (median only). All data was reconstructed using a vendor-neutral algorithm

Medium and large phantom data were only reconstructed using a vendor-neutral algorithm, since results for the small phantom showed the smallest variability between systems for these settings. It can be seen in Fig. 6 that variability of RC between systems increased in larger phantom volumes. Furthermore, smaller sphere diameters showed lower quantitative accuracy (lower RC values) indicating that reliable quantification of small volumes (< 10 ml) in larger (patient) volumes is more challenging.

## Discussion

This study is a considerable step towards standardization of absolute SPECT quantification by investigating the quantitative accuracy of different SPECT/CT systems. The quantitative accuracy of individual SPECT-CT systems was assessed earlier for the GE Discovery NM/CT 670 system [5], the Siemens Symbia Intevo system [44] and the Hermes SUV SPECT quantitative reconstruction algorithm [36]. Although an earlier study by Seret et al. [32] also compared the quantitative capabilities of four SPECT/CT cameras, our study included the current state-of-the-art quantitative SPECT/CT systems that enable absolute quantification that were not available at that time.

Many factors contribute to the uncertainty in quantification even if acquisition protocols are standardized, including VOI outlining methodology, operator variability and activity measurement (dose calibrator uncertainty, cross calibration between dose calibrator, and SPECT/CT system) [45] and in our study also phantom preparation. The median RC in the background compartment was found to be 1.01, which indicated reliable acquisition, reconstruction and analysis. However, for some systems and measurements, the background RC was as low as 0.93 or as high as 1.07. This deviation might of course also influence the sphere RC values and thereby introduce an increase in variability between quantification on different systems. Furthermore, this study showed that the largest contribution for inter-system variation is due to vendor-specific reconstruction settings. Vendor-neutral reconstruction reduced this variation two to threefold (median MAD). It is therefore paramount to harmonize SPECT/CT image reconstructions in a multi-center/multi-vendor setting.

In a clinical setting, it is expected that the variability in quantification between SPECT/CT systems will increase, due to for example patient positioning and patient volume (BMI). To this end, we compared the recovery of the hot spheres in differently sized phantoms on several SPECT/CT systems. Only minor, not clinically relevant differences between the phantoms representing a BMI of 25 and 28 kg/m^2^ were found, while this change in BMI implies a rather significant increase in patient circumference. We therefore expect that for patients with a normal to slightly increased BMI, it is not necessary to take patient circumference into account for quantification. For a high BMI of 47 kg/m^2^ on the other hand, activity could not be recovered for the smaller sphere diameters. This might be explained by the increased attenuation, decreased signal-to-noise ratio, and decreased spatial resolution due to increased source-detector distance in these larger volumes. This means that in patients with a high BMI, quantifying smaller lesions will be more challenging. Using more iterations in the reconstruction of images of larger patients might improve convergence and thereby improve resolution and prevent artifacts, which was also shown for SPECT/CT myocardial perfusion studies by Celler et al. [46]. The effect of increased attenuation could be canceled by an increase in scan time per projection or by increasing patient dose. The impact of scan time and dosage on image quality and image quantification is interesting to investigate further, but this was not within our scope.

The phantom used in this study did not contain lung, air, or bone components. Therefore the results mainly reflect quantification accuracy for soft tissue lesions. Experiments were performed using ^99m^Tc-pertechnetate. This radionuclide is the most widely used in SPECT imaging, and quantification of ^99m^Tc holds potential in for example myocardial perfusion imaging [47], functional lung scanning [48], selective internal radiation therapy (SIRT) of liver tumors [49, 50], quantification in bone lesions [51, 52], and therapy monitoring in locally advanced breast cancer [5]. In addition, since the radiotracer is widely available, it served as a suitable radionuclide to compare absolute quantification performance of SPECT/CT systems.

In the current study, an activity concentration ratio of 1:10 was used between the background and spheres, based on the ratio used for the same phantom in the EARL accreditation program. With lower activity concentration ratios, lower RC values are expected due to partial volume effects.

For one system, matrix size changes were necessary between vendor-specific and vendor-independent reconstructions. With this change, it is uncertain whether the improved inter-scanner variability is due to the vendor-neutral reconstruction algorithm, or to the change in matrix size. It was, however, the aim of our study to assess whether vendor-neutral reconstruction would improve inter-scanner variability. Which underlying parameter caused this improvement was not the goal of our study.

Both vendor dependent as well as vendor-neutral reconstructions showed Gibbs artifacts for all systems, which is a known result of resolution modeling. These artifacts occur especially in phantom reconstructions, with high contrast changes between different structures. In our study, a large contrast change was present between the inside and outside of the spheres. Despite this large contrast change, and its accompanying Gibbs artifact, all systems showed RC_mean_ values approaching unity for larger sphere sizes. When sphere size decreases, the edge ring artifacts will come very close to each other and eventually merge, resulting in a too high activity in the center of the sphere.

In this study, only one vendor-neutral reconstruction algorithm was used. In theory, another reconstruction algorithm, although not commercially available at this moment, could potentially influence the resulting metrics. For the current study, however, our aim was to assess the influence of the reconstruction algorithm on RC measurements which could be assessed by using a vendor-neutral algorithm.

Knowledge gained from this study can be used to assess the absolute quantitative accuracy for other radionuclides as well. This can serve as input for a standardization program for absolute SPECT quantification which can be used to improve sophisticated clinical dosimetry in radionuclide therapy studies, especially in a multi-center setting.

## Conclusion

This study shows that absolute SPECT quantification is feasible in a multi-center and multi-vendor setting. With center-specific reconstructions, variability between systems was 0.01–0.20 and 0.03–0.28 (MAD) for RC_mean_ and RC_max_, respectively. Standardized reconstruction decreases this variability to 0.02–0.05 and 0.04–0.11. Variation between centers is mainly caused by the use of different reconstruction algorithms and/or settings. Patient size showed to be relevant for quantification, as it was observed that high patient volume (BMI 47 kg/m^2^) resulted in an increased variability among systems and impeded quantification of small lesions (< 10 ml). Close agreement between vendors and centers is key for reliable multi-center dosimetry and quantitative biomarker studies. This study serves as a first step towards a vendor-independent standard for absolute quantification in SPECT/CT.

## Supplementary information

**Additional file 1: Table S1.** Settings of low dose CT protocols used for attenuation correction. **Table S2.** Cross-calibration protocols for dose calibrators to SPECT/CT system according to vendor recommendations.

## Data Availability

The datasets used and/or analyzed during the current study are available from the corresponding author on reasonable request.

## Abbreviations

^111^In: Indium-111
^131^I: Iodine-131
^177^Lu: Lutetium-177
^90^Y: Yttrium-90
^99m^Tc: Technetium-99m
BMI: Body mass index
CF: Calibration factor
CT: Computed tomography
DEW: Dual energy window
EARL: EANM Research Ltd.
FDG: Fluorodeoxyglucose
LEHR: Low-energy high-resolution
MAD: Median absolute deviation
OSEM: Ordered subset expectation maximization
PET: Positron emission tomography
PSF: Point spread function
RC: Recovery coefficient
RC_max_: Max recovery coefficient
RC_mean_: Mean recovery coefficient
ROI: Region of interest
SIRT: Selective internal radiation therapy
SPECT: Single-photon emission computed tomography
TEW: Triple energy window
VOI: Volume of interest
