# Supplementary material for: Towards standardization of absolute SPECT/CT quantification: a multi-center and multi-vendor phantom study
Source: EJNMMI Phys. 2019 Dec 26;6:29. doi: 10.1186/s40658-019-0268-5 (PMC6933042; doi:10.1186/s40658-019-0268-5)
Supplement: Supplementary file 1 — Additional file 1: Table S1. Settings of low dose CT protocols used for attenuation correction. Table S2. Cross-calibration protocols for dose calibrators to SPECT/CT system according to vendor recommendations. [file 40658_2019_268_MOESM1_ESM.docx]

**Supplemental data**

Table S1: Settings of low dose CT protocols used for attenuation correction

| System | Discovery NM/CT 670 Pro | Precedence 6 | Symbia Intevo 6 | Symbia T16 |
| --- | --- | --- | --- | --- |
| Detector rows | 24 | 6 | 6 | 24 |
| Tube voltage [kVp] | 100 | 120 | 130 | 110 |
| Tube current time product [mAs] | 30 (effective) | 30 (effective) | 15 (reference) | 30-37 (effective) |
| Slice thickness [mm] | 1.25 | 6x3 | 2.0 | 5.0 |
| Reconstruction kernel | Standard | Detail (D) | B31s | B08s medium sharp |
| Reconstructed slice thickness [mm] | 4.0 | 3.3 | 3.0 | 5.0 |
| Reconstruction FOV [mm] | 500 | 600 | 650 | 500 |

Table S2: Cross-calibration protocols for dose calibrators to SPECT/CT system according to vendor recommendations

| System | System/Center specific cross-calibration method |
| --- | --- |
| Discovery NM/CT 670 Pro | System sensitivity, measured using a Petri dish filled with 90 MBq ^99m^Tc-pertechnetate [3] |
| Precedence 6 | Cylindrical phantom with diameter of 20 cm filled with 7 – 10 kBq/ml ^99m^Tc-pertechnetate |
| Symbia Intevo 6 | NIST trace­able calibration using a ^57^Co source (Calibrated Sensitivity Source (CSS).  Verified with a cylindrical phantom with diameter of 20 cm |
| Symbia T16 | Cylindrical phantom with diameter of 20 cm filled with 7 – 10 kBq/ml ^99m^Tc-pertechnetate |
| All, Hermes SUV SPECT | Cylindrical phantom with diameter of 20 cm filled with 7 – 10 kBq/ml ^99m^Tc-pertechnetate |
